# Experimental Study of Retention on the Combination of Bifidobacterium with High-Intensity Focused Ultrasound (HIFU) Synergistic Substance in Tumor Tissues

**DOI:** 10.1038/s41598-019-42832-4

**Published:** 2019-04-23

**Authors:** Xuan Gao, Wenjuan Zou, Binglei Jiang, Die Xu, Yong Luo, Jie Xiong, Sijing Yan, Yaotai Wang, Yu Tang, Chun Chen, Huanan Li, Hai Qiao, Qi Wang, Jianzhong Zou

**Affiliations:** 1 State Key Laboratory of Ultrasound Engineering in Medicine Co-Founded by Chongqing and the Ministry of Science and Technology, Chongqing Collaborative Innovation Center for Minimally-invasive and Noninvasive Medicine, College of Biomedical Engineering, Chongqing Medical University, Chongqing, 400016 China; 2 Chongqing Traditional Chinese Medicine Hospital, Chongqing, 400021 China

**Keywords:** Targeted therapies, Outcomes research

## Abstract

High intensity focused ultrasound (HIFU) has been recently regarded to be a new type of technique for non-invasive ablation of local tumors and HIFU synergists could significantly improve its therapeutic efficiency. The therapeutic efficiency of HIFU is greatly limited by the low retention of HIFU synergists in the target area and short residence time. This study aimed to explore a method to increase the deposition of HIFU synergists in tumors. Cationic lipid nanoparticle can be used to enhance the HIFU ablation effect, but there is still a problem for it that the deposition amount in the tumor tissue is small and the residence time is short. Bifidobacterium is highly biosafe and can be selectively colonized in the hypoxic zone of tumor tissue. Cationic lipid nanoparticles can be observed *in vitro* by attachment to bifidobacterium by electrostatic adsorption. And the effect of the proliferation of bifidobacterium in tumor tissues on the retention amount and retention time of cationic lipid nanoparticles *in vivo* was evaluated. Results showed that the cationic lipid nanoparticles were linked to the surface of Bifidobacterium effectively *in vitro*, while *in vivo*, the retention amount and retention time of cationic lipid nanoparticles could be increased by Bifidobacterium in tumor tissues, which provided a new method for improving the therapeutic efficiency of HIFU.

## Introduction

Malignant tumors are serious diseases that significantly threaten human health. High-intensity focused ultrasound (HIFU) is an emerging non-invasive treatment technique which can focuses low-energy ultrasound on the targeted areas to treat the lesion via cell necrosis caused by the instant heat effect, cavitation effect, mechanical effect^1–4^. In the past decades, HIFU has been widely recognized for its safety, efficiency in the treatment of solid tumors and non-neoplastic disease^5–10^. However, there are series of challenges in the treatment process. For example, when treating deep tissue, the energy of the ultrasound decreases exponentially with increasing distance^11^. In addition, the flow of blood in the tissue also eliminates some of the heat and reduces the energy deposition in the target area, which greatly reduces the therapeutic efficiency of HIFU^12^. Therefore, increasing the output power and prolonging the treatment time are the main methods for improving the therapeutic efficiency of HIFU. However, these methods cause damage to normal tissues surrounding the acoustic channel, causing serious side effects^13–15^. Given all these reasons, synergistic agents such as lipiodol and hydroxyapatite were used to alter the acoustic environment of the tissue (TACE), which can improve the therapeutic effect of HIFU by increasing the deposition of energy^16^. However, these synergists stay in the body for a short period of time and are present in both tumors and normal organs, resulting in susceptibility to acoustic channel tissue damage when treated with HIFU. Although the cationic lipid nanoparticle with liquid fluorocarbon can increase the ultrasonic ablation effect because of its liquid-gas phase change characteristics, but its further clinical application is limited because of its short residence time in tumor tissue and small retention amount^17^.

Bifidobacterium (BF) is a common probiotic in the intestine. It has been proven to be safe, non-toxic, no pathogenesis and immune side effects to the host, and can exert antitumor effects to a certain extent^18–20^. Numerous studies have shown that with the growth of solid tumors, a large number of anaerobic areas appear in tumors due to ischemia and hypoxia. The facultative anaerobic properties of bifidobacterium make it easy to concentrate and multiply in the anaerobic zone of the tumor after entering the blood, indicating tumor targeting^21,22^. Because it tends to be an anaerobic environment rather than a specific receptor for a tumor or tissue. Thus, solid tumors of different sites and different tissue sources can be targeted. According to Kimura’s report, bifidobacterium could localize and proliferate in the hypoxic zone of tumor tissue. In his study, a large number of bifidobacterium was detected in tumors 48–96 hours after tail vein injection, but not in normal organs^22^. Additional studies have shown that bifidobacterium can be used as carriers carrying genes and contrast agents to reach tumor tissues for tumor imaging and therapeutic purposes^23–25^.

To overcome the problem of low accumulation and short residence time of cationic lipid nanoparticles in tumor tissues. This study investigated the effect of colonization of bifidobacterium on the retention amount and retention time of cationic lipid nanoparticles in tumor tissues, which provided a possibility to further improve the efficiency of HIFU treatment.

## Results

### Characterization of PEGylated Cationic lipid nanoparticles(CL-NPs) and Bifidobacterium(BF) longum ATCC 15707

**Figure 1:**
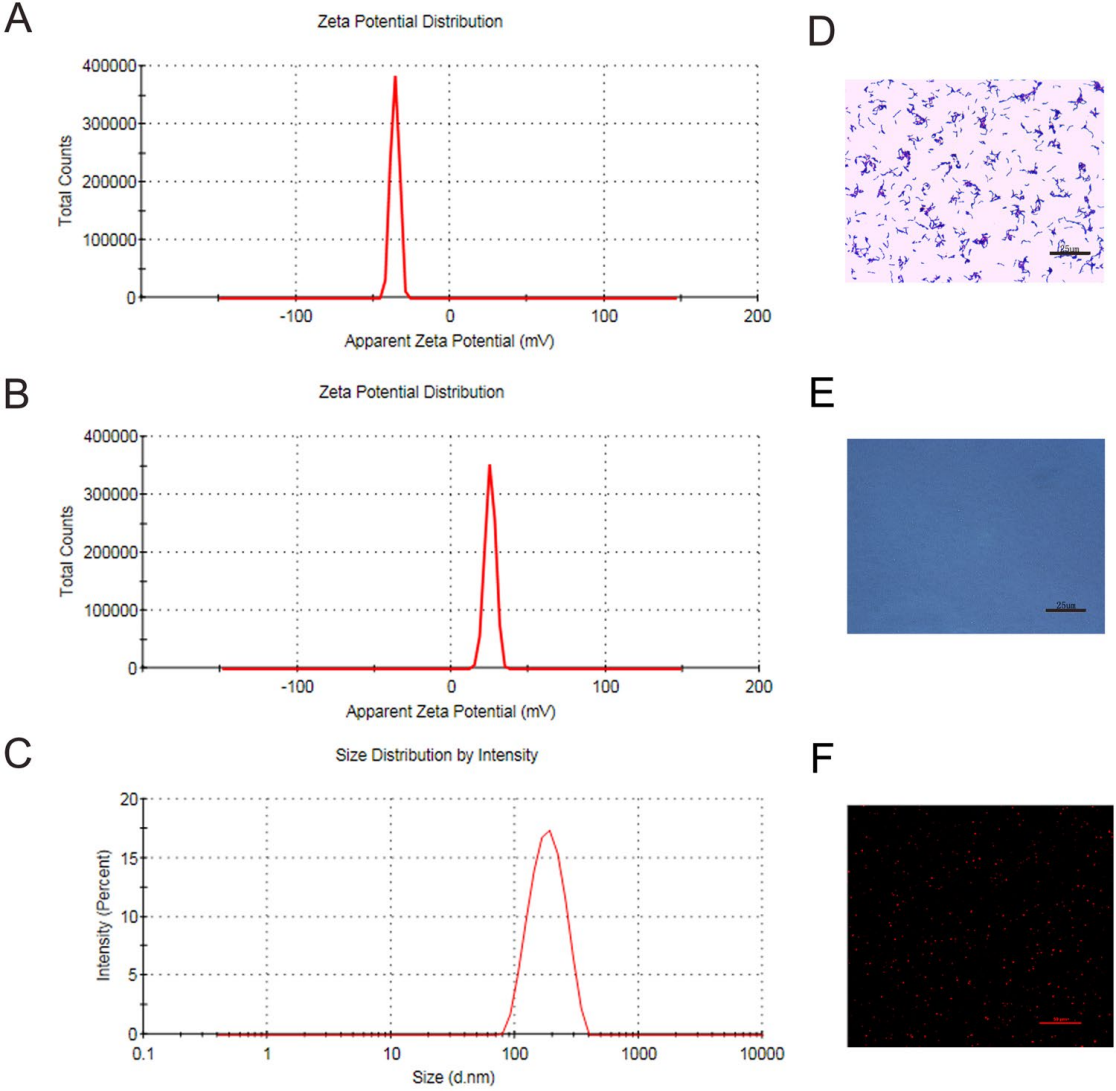
(**A**) Surface potential of bifidobacteria longum ATCC15707. (**B**,**C**) Surface zeta potential and particle size distribution of CL-NPs. (**D**) Optical microscope image of Gram stain of bifidobacteria longum ATCC 15707 (400 × magnification). (**E**) Optical microscope image of CL-NPs(400 × magnification). (**F**) CLSM image of CL-NPs(800 × magnification).

CL-NPs showed a uniform suspoemulsion, when observed visually. The nanodroplets were visualized under an optical microscope and a confocal laser-scanning microscope (CLSM) (Fig. 1E,F). The prepared CL-NPs had an average diameter of 280 ± 60 nm (PDI = 0.309) (Fig. 1C) and an average surface zeta potential of +38 mV (Fig. 1B). The average surface zeta potential of Bifidobacterium was −29 mv (Fig. 1A).

### *In vitro* experiments

**Figure 2:**
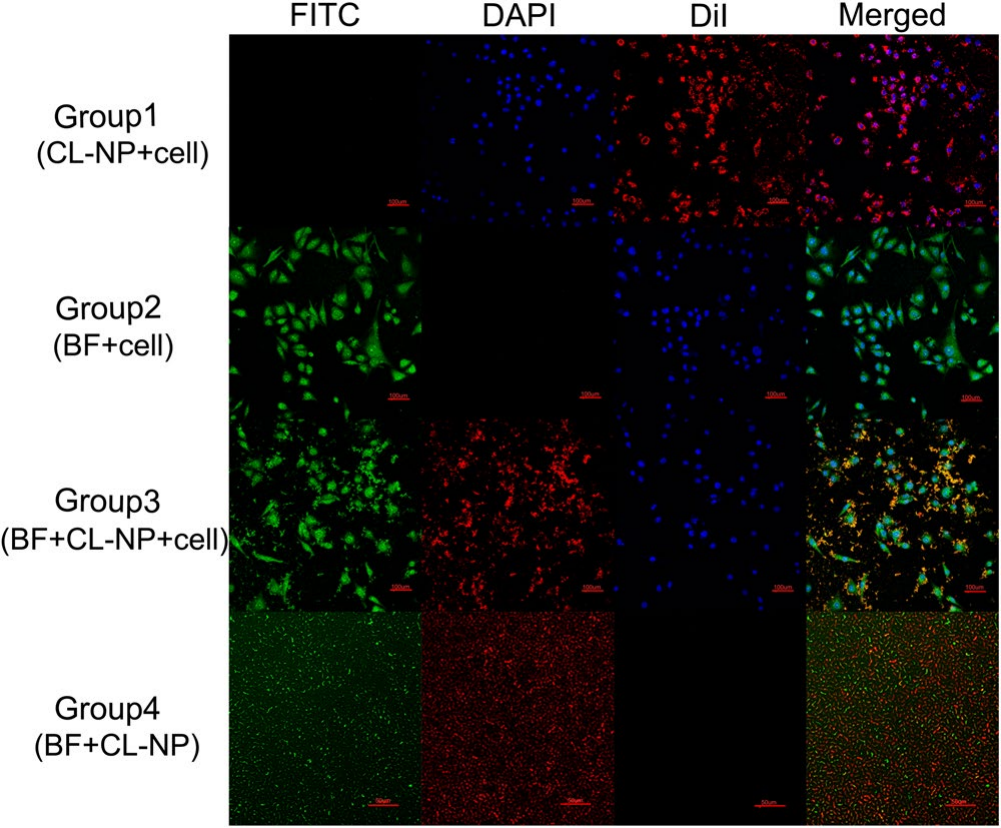
Group1, CLSM images of MDA-MB-231 cells co-incubated with CL-NPs (800 × magnification). Group2, CLSM images of MDA-MB-231 cells co-incubated with BF (800 × magnification). Group3, MDA-MB-231 cells first co-incubated with BF and then co-incubated with CL-NPs (800 × magnification). Group4, CLSM images of CL-NPs co-incubated with BF (400 × magnification). Blue, DAPI-stained nuclei; green, FITC-labeled bifidobacteria and cell membrane; red, DiI-labeled CL-NPs.

To accurately observe the connection between Bifidobacterium(BF) and Cationic lipid nanoparticles(CL-NPs), we observed cells via CLSM. The experiments were labeled with triple fluorescence (red fluorescence from DiI labeled CL-NPs, Green fluorescence from FITC-labeled bifidobacterium, and Blue fluorescence from DAPI-labeled cell Nuclear). In group 1 (MDA-MB-231 cells + CL-NP), many red fluorescently labeled CL-NPs were observed around the cell membrane and cytoplasm (Fig. 2). In group 2 (MDA-MB-231 cells + BF), we did not observe that FITC-labeled bifidobacterium was attached to the periphery of the cell membrane and cytoplasm. While the cell membrane was stained with green fluorescence by the residual FITC in the bifidobacterium solution (This phenomenon was unavoidable because the cell membrane is easily stained by FITC). In group 3 (MDA-MB-231 cells + CL-NP + BF), we observed significant yellow fluorescence (superimposition of green fluorescence from FITC-labeled bifidobacterium and red fluorescence from DiI-labeled CL-NPs) around the cell membrane, and the cell membrane was stained green by FITC remaining in the FITC-labeled bifidobacterium solution. In group 4 (CL-NP + BF), many CL-NPs represented by red fluorescence adhered to the periphery of BF represented by green fluorescence. The above results indicated that DiI-labeled CL-NPs could not only adhere to the periphery of bifidobacterium, but also adhere to the periphery of the cell membrane, laying a foundation for experiments *in vivo*.

### Retention efficiency of Bifidobacterium(BF) on PEGylated Cationic lipid nanoparticles(CL-NPs) *in vivo*

**Figure 3:**
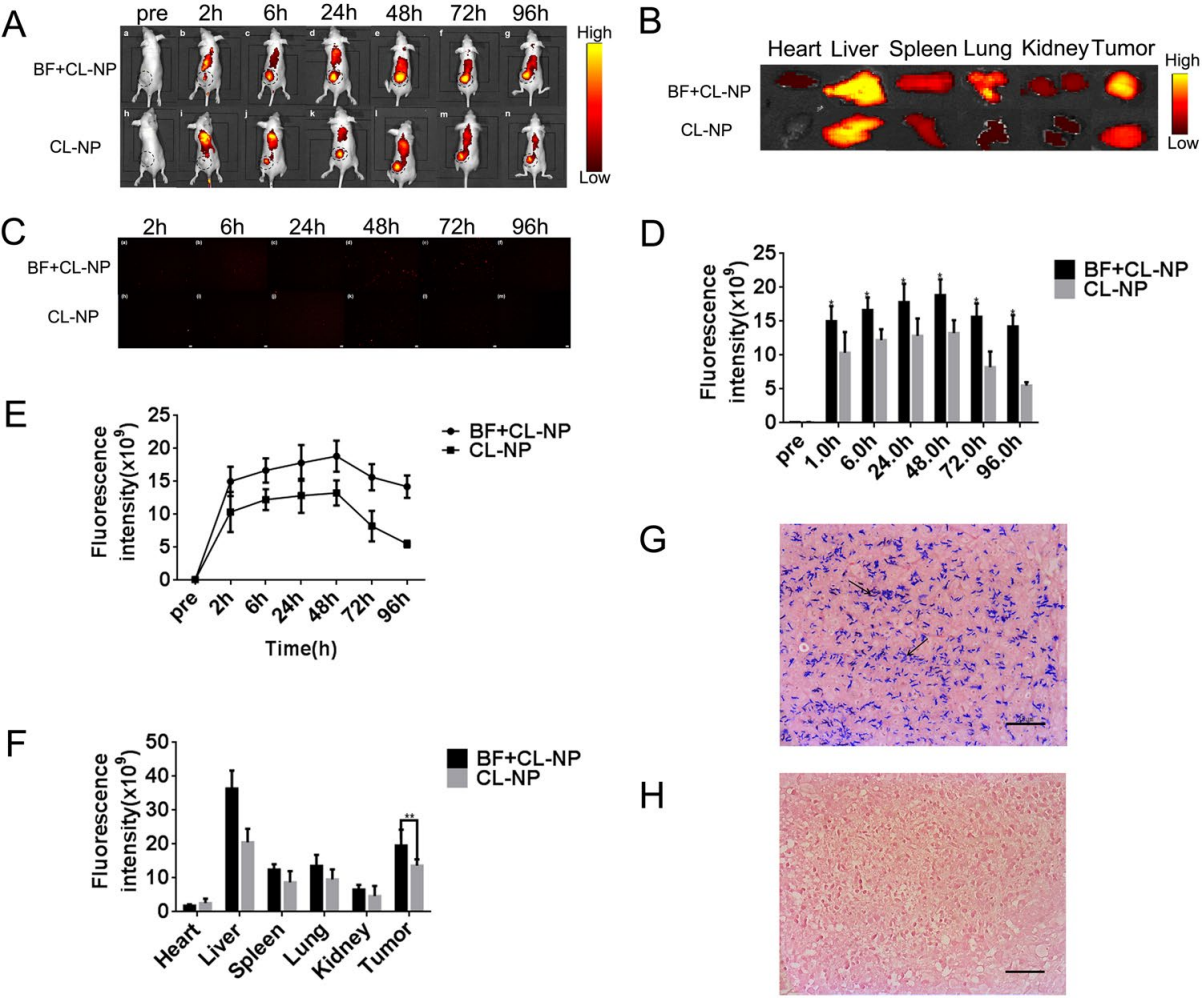
(**A**) FLI images of mice at different time points (2, 6, 24, 48, 72, 96 h) after injection of DiR-labeled CL-NPs in the BF + CL-NP group and CL-NP group, the black dotted circle marks the tumor nodules. (**B**)Isolated FLI images of major organs and tumors in the BF + CL-NP and CL-NP groups 48 hours after injection of DiR-labeled CL-NPs in the tail vein. (**C**) FLI images of ultrathin sections of tumor tissue at different time points (2, 6, 24, 48, 72, 96 h) after injection of Dil-labeled CL-NPs in the tail vein. (**D**) The fluorescence intensity in tumors measured at different time points (2 h, 6 h, 24 h, 48 h, 72 h, 96 h) after injection of DiR-labeled CL-NPs in the tail vein (*P < 0.05). (**E**) The mean fluorescence intensity of tumor tissues in the CL-NP + BF group and the CL-NP group changed with time, after the tail-injection of DiR-labeled CL-NPs. (**F**) The mean fluorescence intensity of major isolated organs after tail vein injection of CL-NPs for 48 h (**P < 0.01). (**G**,**H**) The Gram staining of tumor tissues of tumor-bearing mice in CL-NP + BF group and CL-NP group (400 × magnification).

To further validate the retention efficiency of bifidobacteriaum in CL-NPs in tumor tissues, small animal live fluorescence imaging (FLI) was performed with MDA-MB-231 tumor-bearing nude mice. In the BF + CL-NP group, strong red fluorescence was observed in the tumor area 1–96 h after injection of the DiR-labeled CL-NPs. Moreover, the fluorescence intensity and range decreased slowly after reaching a peak at 48 h (Fig. 3A,D,E). In the CL-NP group, relatively weak red fluorescence was observed in the tumor area 1–96 h after injection of the DiR-labeled CL-NPs. In addition, the fluorescence intensity and range rapidly decreased after reaching a peak at 48 h (Fig. 3A,D,E). The fluorescence intensity of tumor tissue in the BF + CL-NP group was greater than that in the CL-NP group throughout the observation period. (*P < 0.05; Fig. 3A,D,E). After injection of CL-NPs for 48 h, the fluorescence intensity of tumors in CL-NP + BF group decreased  slower than CL-NP group (Fig. 3E). After injection of CL-NPs for 48 h, the FLI of the isolated organs showed that the fluorescence intensity of the tumor area of the BF + CL-NP group was greater than that of the CL-NP group (**P < 0.01; Fig. 3B,F). The fluorescence intensity of normal tissues of the BF + CL-NP group was stronger than that of the CL-NH group at 1 h, 6 h, 24 h, 48 h, 72 h, and 96 h (Fig. 3F). Ultrathin sections of tumor tissue showed that the concentration of CL-NPs represented by red spots in the BF + CL-NP group was greater than that of the CL-NP group throughout the observation period and reached the maximum number at 48 hours (Fig. 3C). Gram staining of the tissue sections showed that there was many bifidobacterium (BF) scattered distribution in the tumor tissues of the BF + CL-NP group, and no bifidobacterium was found in the tumor tissues of the CL-NP group (Fig. 3G,H). The above phenomenon might be caused by bifidobacterium in tumor tissues, which increased the deposition amount and residence tine of CL-NPs in tumor tissues.

### *In vivo* HIFU ablation

**Figure 4:**
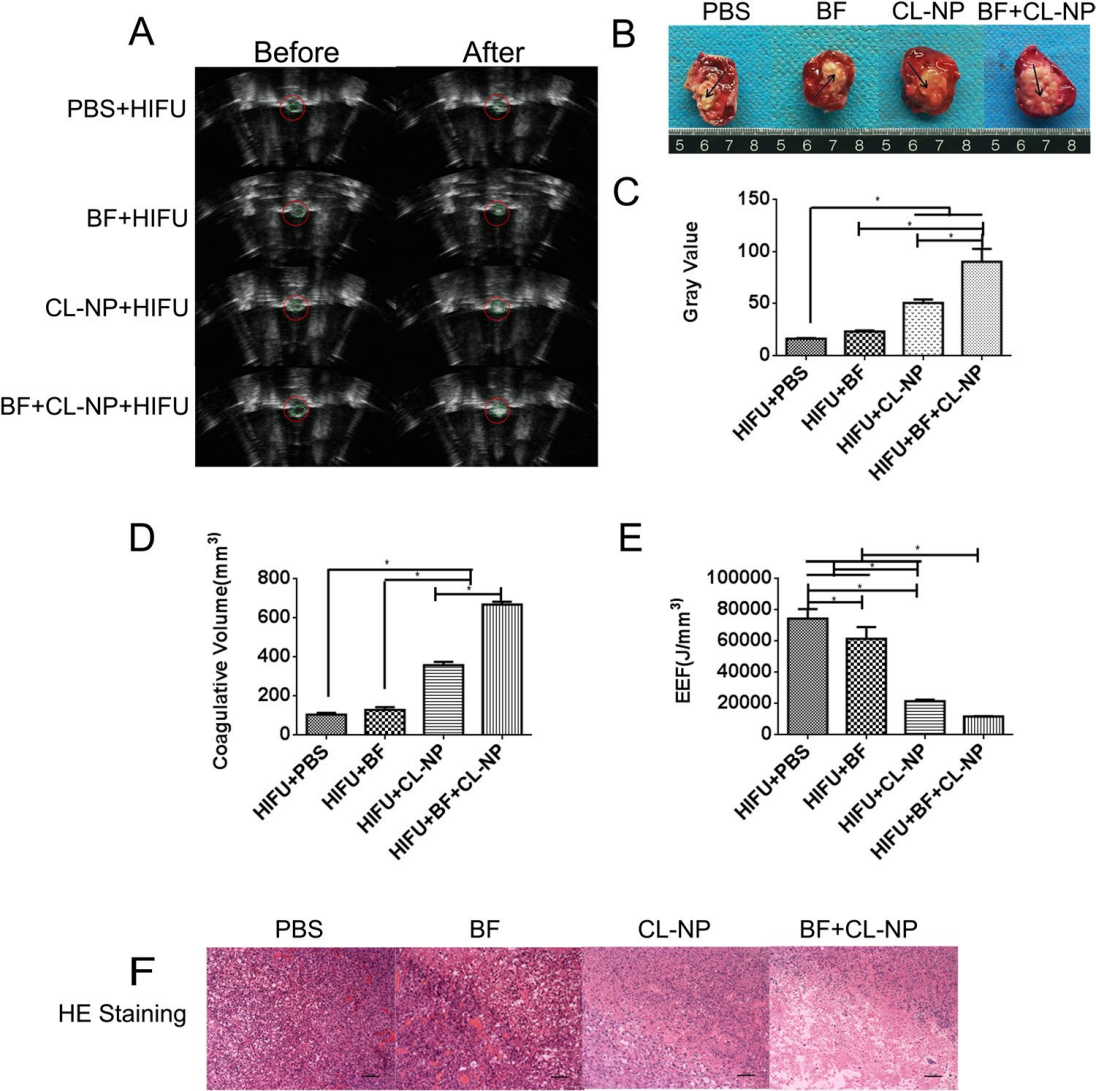
(**A**) *In vivo* ultrasound imaging of tumor tissues (red circle) before HIFU ablation and after ablation: HIFU + PBS, HIFU + BF, HIFU + CL-NP, HIFU + BF + CL-NP. (**B**) TTC staining of each group of tumor tissues after HIFU irradiation, the necrotic tissue appears gray and the unablated tumor is red. (**C**) The comparison of gray values of tumor tissues in each group after HIFU irradiation. (**D**) The comparison of coagulative necrotic volume of tumor tissues in each group after HIFU irradiation. (**E**) The comparison of EEF values of tumor tissues in each group after HIFU irradiation. (**F**) HE staining of tumor tissues of each group after HIFU irradiation (100 × magnification).

The role of Bifidobacterium(BF) in retaining Cationic lipid nanoparticles(CL-NPs) in the tumor was further verified in four groups (I: HIFU + PBS, II: HIFU + BF, III: HIFU + CL-NP, IV: HIFU + BF + CL-NP). HIFU was used to ablate the tumor tissue under specific ablation parameters (sound power 150 w, exposure time 5 s). Immediately after HIFU irradiation, varying degrees of grayscale and echo intensity changes were clearly observed within the focal volume (Fig. 4A). The tumor tissues of group I, II, III, and IV showed varying degrees of gray value changes after HIFU irradiation. The changes in gray value of group IV were significantly greater than those in group I, group II and group III (Fig. 4C, *P < 0.05). The gray value changes of Group III were greater than group I and group II (Fig. 4C, *P < 0.05). When the HIFU ablation volume was compared, we observed that the coagulative necrosis volume of group IV was the largest, followed by group III, and the necrotic volume of group III was greater than that of group II and group I (*P < 0.05, Fig. 4B,D). The EEF value of Group IV was much smaller than the other groups(*P < 0.05, Fig. 4E). The EEF values of group I and group II were followed by group III (*P < 0.05, Fig. 4E). In summary, the HIFU ablation effect of group IV was greater than the other groups, which indicated that Bifidobacterium could effectively retain cationic lipid nanoparticles *in vivo*, exhibiting a strong synergistic effect. HE staining showed varying degrees of damage within the ablation area. The morphology of the tumor cell membrane of group I was slightly damaged and the nucleus morphology was intact. Severe cell damage and lysed cell membranes and nuclear fragmentation were easily observed in the coagulative necrotic areas of group III and group IV. Damages in group IV were more serious than group III (Fig. 4F). The images further demonstrated that more cationic lipid nanoparticles could be retained by Bifidobacterium in the tumor tissue, resulting in more efficient HIFU ablation.

## Discussion

The PEGylated CL-NPs prepared in this study was 280 nm, which readily penetrated into the tumor tissue through the vascular endothelium. In particular, liposome polyethylene glycol (PEG: polyethylene glycol) can effectively shield the positive charge on the surface of the cation, thereby reducing the non-specific adsorption of CL-NPs to certain biological macromolecules in the blood^26,27^. In addition, PEGylation of liposomes not only overcomes the key problems of rapid elimination by the mononuclear phagocytic system (MPS) in the systemic circulation, but also possible to increase the passive accumulation by extending the circulation of the nanoparticles, thereby minimizing the side effects of the nano-reagent^28,29^. The core of CL-NPs is hydrophobic liquid perfluorohexane (PFH), which undergoes liquid-gas phase transition when the external pressure is reduced to the gasification threshold or the temperature is raised above the boiling point^30^. HIFU irradiation can increase the ablation effect, which would be confirmed in subsequent experiments.

The surface of CL-NPs prepared in this study was positive potential, and the surface of bifidobacterium was negative potential. Therefore, CL-NPs could adhere to the surface of the negatively charged bifidobacterium and the surface of the negatively charged cell membrane by electrostatic adsorption. CLSM showed that both the surface of bifidobacterium and the surface of cell membrane could adhere to a large amount of CL-NPs by electrostatic adsorption, but the number of CL-NPs on the surface of bifidobacterium was larger than the number of CL-NPs on the surface of cell membrane. The specific mechanism of this phenomenon is still unclear, but this phenomenon lays a foundation for the role of bifidobacterium retaining cationic lipid nanoparticles in tumor tissues.

The results of FLI *in vivo* showed that the fluorescence intensity of the tumor area in the CL-NP + BF group was greater than that in the CL-NP group throughout the observation period, and the fluorescence intensity of the tumor tissues reached a maximum at 48 h. After 48 h, the fluorescence metabolism rate of the tumor area in the CL-NP group was significantly higher than that in the CL-NP + BF group. Meanwhile, the results from the ultrathin section of tumor tissue also displayed the accumulation of CL-NPs in the CL-NP + BF group were more than that in the CL-NP group at each moment. There would be several reasons for this phenomenon. Firstly, CL-NPs can not only adhere to the surface of the cell membrane, but also the surface of bifidobacterium in the tumor tissue can adhere to a certain amount of CL-NPs, which may lead to an increase in the retention of CL-NPs in the tumor tissue. Secondly, bifidobacterium proliferates in tumor tissues, which can activate macrophages and increase the phagocytic function of macrophages^31,32^. Because endocytosis is the primary mechanism by which liposomes enter cells and activated macrophages may engulf more CL-NPs, thus enhancing the deposition amount of CL-NPs in tumor. Thirdly, the proliferation of bifidobacterium in tumors can increase NO levels, which can enhance the permeability of blood vessels and the EPR effect of blood vessels^33,34^. Therefore, the amount of CL-NPs that enter the tumor tissue cross the vascular endothelium may increase, enhancing the deposition amount of CL-NPs in the tumor tissues. Fourthly, the proliferation of bifidobacterium in tumor tissues may change the microenvironment of tumor tissues, and the metabolic rate of CL-NPs may decrease, then the retention time of CL-NPs in tumors was prolonged. Finally, the metabolism of complexes of bifidobacterium and CL-NPs in tumors may be slower than the metabolism of CL-NPs on the surface of cell membranes, which may increase the retention time of CL-NPs in tumor tissues.

In the HIFU irradiation experiment, the synergistic effect of bifidobacterium in tumor tissues combined with CL-NPs on HIFU treatment was investigated, involving coagulative necrotic volume, gray value change and energy efficiency factor (EEF). Compared with PBS group, the volume of coagulative necrosis in the target area and the gray scale value alteration of CL-NP group was larger under the same condition, exhibiting a strong synergistic effect. The result indicated that CL-NPs has the effect of increasing HIFU to ablate tumors, which was the same as the previous studies. Compared with the other three groups, under the same conditions, after HIFU irradiation, the BF + CL-NP group had the largest coagulative necrosis volume and the gray scale value alteration, showing the strongest synergistic effect. This phenomenon could be explained by the following reasons. First of all, the acoustic environment of tumor tissues may be changed by the proliferation of bifidobacterium, which may increase the deposition of ultrasound energy and then the efficacy of HIFU is improved. Secondly, the increased deposition of CL-NPs may reduce the cavitation threshold of tumor tissues, increasing the ultrasound energy deposition, and then therapeutic effect of HIFU was increased^35–37^. Pathological examination showed that the degree of destruction of tumor cells in the target area of each group was different, and the tumor cells in the BF + CL-NP group were the most severely damaged. The possible mechanism for this phenomenon is that under the action of mechanical force caused by ultrasound, CL-NPs in the tumor can exert alternating pressure on the cells to squeeze and expand, causing the tumor cells to rupture. The deposition of CL-NPs in tumors were increased, and the degree of rupture of tumor cells may be more serious, which strongly supported the conclusion above. In this study, the smallest EEF value was observed in the BF + CL-NP group, indicating that bifidobacterium combined with CL-NPs in the tumor required the least ultrasound energy to treat a unit volume of lesions, with the least possible damage to the organism. These findings suggest that bifidobacterium combined with CL-NPs in tumor tissue may increase the deposition amount of CL-NPs in the tumor, and then the ablation effect of HIFU was increased. However, the specific mechanism needs further research and exploration. This method is consistent with the non-invasive principle of HIFU treatment and demonstrates the effectiveness of bifidobacterium in combination with CL-NPs in the treatment of HIFU.

We report for the first time that colonization of bifidobacterium in tumor tissue increases the deposition and retention duration of HIFU synergistic substance in tumor tissues. This study improved the shortcomings of traditional HIFU synergistic substances in tumors, and increased the deposition of CL-NPs in tumors, which improved the therapeutic efficiency of HIFU to some extent. In addition, this study has the potential to reduce the side effects caused by repeated and long time traditional HIFU treatment, which provides great hope for further improving the development of microinvasive technology.

## Methods

### Preparation of PEGylated Cationic lipid nanoparticles(CL-NPs)

A certain mass of lipid [5 mg DPPC, 2 mg DSPE-PEG (2000)-Amine and 2 mg DC-cholesterol(Avanti Polar Lipids, Inc, Alabaster, AL, USA)] were mixed together and dissolved into 10 mL trichloromethane(CHCl3) (Fisher Scientific, Waltham, MA, USA). The solution was moved to the rotary evaporator (Yarong Inc, Shanghai, China) at 50 °C to remove the organic solvent and form a thin lipid film. One hour later, the resulting thin lipid films was hydrated in 2 mL Double steaming water forming a translucent opalescent suspension. Then 100 µl of Perfluorohexane (PFH) was dropwise into suspension after which the suspension was emulsified in an ice bath using a sonicator (Sonics &Materials Inc., Newtown, CT, USA) with power of 130 W for 5 min (5 s on and 5 s off), then the prepared cationic liquid nanoparticles (CL-NPs) were harvested. The nanoemulsions were centrifuged at 6000 rpm for 5 min and then washed in Double steaming water to wipe off disasociated lipids and PFH. The centrifugation and washing process were repeated three times. Finally, the prepared CL-NPs were stored at 4 °C for later use. To prepare fluorescent nanodroplets, DiI (1 mg) or DiR (1 mg) fluorescent dye were added to the lipid solution and aluminum foil papers was used to prevent light exposure.

### Bifidobacteria(BF) culture

Bifidobacterium longum (ATCC 15707) was cultured in MRS broth for 24 h under anaerobic conditions at 37 °C. The Bifidobacterium was harvested by centrifuging at 1000 rpm for 10 min at 4 °C. The Bifidobacterium was diluted with phosphate buffered saline (PBS) (pH 7.4), and the concentration of bifidobacterium was adjusted to about 2 × 10^8^ bacilli/ml.

### Characterization of PEGylated Cationic lipid nanoparticles(CL-NPs) and Bifidobacteria(BF)

Fuorescence was observed by confocal laser scanning microscope (CLSM, Nikon A1, Japan). The particle size distribution and zeta potential were measured by Malvern Zetasizer Nano ZS (Malvern Instruments, UK).

### Cell line and cell culture

The human breast cancer MDA-MB-231 cell line was obtained from the Institute of Ultrasound Imaging, Chongqing Medical University (Chongqing, China). The cells were cultured in dulbecco’s modified eagle medium(DMEM) medium supplemented with 10% fetal bovine serum (FBS) and 1% penicillin/streptomycin. Then the cells were cultured in an incubator with a humidified atmosphere containing 5% CO_2_ at 37 °C. The cells were used for the experiments when they were in a logarithmic growth phase.

### Animals and model establishment

All animals (female BALB/c nude mice: ~20 g, 4–6 weeks old) were purchased from the Chongqing Experimental Animal Center Medical University. All experiments and procedures were approved by the Institutional Animal Care and Use Committee of Chongqing Medical University and all methods were performed in accordance with the relevant guidelines and regulations. For the establishment of the tumor model, each nude mouse was subcutaneously injected with 1 × 10^6^ MDA-MB-231 cells suspended in 200 µL PBS solution in the left flank.

### *In vitro* experiments

Culture dishes were divided into four groups: group1: MDA-MB-231 cells + CL-NP, group 2: MDA-MB-231 cells + BF, group 3: MDA-MB-231 cells + BF + CL-NP, group 4: BF + CL-NP. MDA-MB-231 cells were seeded at a density of 1 × 10^4^ cells per dish in the designated group 1, 2, and 3 dishes, and cells were not seeded in the fourth group. After 24 h, the cells were treated based on the different groups after forming a stable monolayer in each dish. Group 1,300 μl DiI-labeled CL-NPs (1 mg/ml) were added for 10 min. Group 2,100 µL FITC-labeled bifidobacterium(1 × 10^6^ bacilli/ml) were added for 10 min. Group 3,100 µL of FITC-labeled bifidobacterium (1 × 10^6^ bacilli/ml) were added for 10 min and then 300 μL of DiI-labeled CL-NPs (1 mg/ml) were added for 10 min. Group 4,100 μL of FITC-labeled bifidobacterium (1 × 10^6^ bacilli/ml) were added and then 300 μL of DiI-labeled CL-NPs (1 mg/ml) were added for 10 min. Groups 1, 2, and 3 were washed three times with sterile PBS, fixed with 4% paraformaldehyde (1 ml) for 30 min, and incubated with DAPI (1 µg/mL, 200 µl) for 20 min. After administration of paraformaldehyde and each fluorescent dye, dishes of group 1, 2 and 3 were washed three times with PBS, and group 4 was diluted 200-fold. Finally, all the dishes were wrapped in aluminum foil to avoid light and sent for CLSM (Nikon A1, Japan).

### Retention efficiency of Bifidobacterium(BF) on Cationic lipid nanoparticles(CL-NPs) *in vivo*

Two weeks after tumor inoculation, 30 mice bearing xeno-graft MDA-MB-231 tumor (0.5 cm in diameter) were randomly divided into two groups, 15 per group (group A: BF + CL-NP, group B: CL-NP) for fluorescence imaging (FLI). All experiments and procedures were approved by the Institutional Animal Care and Use Committee of Chongqing Medical University and all methods were performed in accordance with the relevant guidelines and regulations. The tumor-bearing mice of group A were injected with 200 µl of sterile PBS and tumor-bearing mice of group B were injected with 200 µl of bifidobacterium at a concentration of 1 × 10^8^ bacilli/ml through the tail vein. After 7 days, all mice were anesthetized with barbital (6.25 ml/kg) via intraperitoneal injection of 1% sodium pentobarbital, and then 200 µl of DiR-labeled CL-NPs(1 mg/ml) were injected through the tail vein. The dose of PFH was 100 µl/kg relative to the body weight of the mice, which was lower than the reported dose of 126 µl/kg. Mice were observed using the FLI system (CRi Inc, Woburn, MA, USA) 2 h, 6 h, 24 h, 48 h, 72 h and 96 h after injection of DiR-labeled CL-NPs, with excitation and emission wavelengths of 748 nm and 780 nm. The fluorescence intensity of the tumor area was measured after intravenous injection of DiR-labeled CL-NPs for 2 h, 6 h, 24 h, 48 h, 72 h, and 96 h. 48 hours after the injection of DiR-labeled CL-NPs, 5 tumor-bearing mice were randomly selected from each group, and the organs (heart, liver, spleen, lung, kidney and tumor nodules) of the tumor-bearing mice were extracted for *ex vivo* FLI. The average value was used to record the fluorescence intensity. The fluorescence intensity was proportional to the concentration of CL-NPs. Tumor-bearing mice were sacrificed after FLI *in vivo*. Tumors and normal tissues were then excised, fixed in 10% formalin solution, sectioned with paraffin and stained with Gram stain, which was used to demonstrate that bifidobacterium could target tumors.

Another 36 tumor-bearing mice were randomly divided into two groups (group a: BF + CL-NP, group b: CL-NP), and the experiments were started when the tumor diameter was 0.5 cm. The nanoemulsion were dyed with DiI and the rest of the treatments were the same as above. Three tumor-bearing mice were sacrificed at 2 h, 6 h, 24 h, 48 h, 72 h and 96 h after injection of DiI-labeled CL-NPs. Tumor tissues were extracted, cut into ultrathin sections (6 μm), and CL-NPs in tumor tissues were observed by fluorescence microscopy (CKX41; Olympus).

### *In vivo* HIFU ablation

When the tumor diameter was 1 cm, 40 tumor-bearing mice were randomly divided into four groups, 10 per group (I: HIFU + PBS, II: HIFU + BF, III: HIFU + CL-NP, IV: HIFU + BF + CL-NP). All experiments and procedures were approved by the Institutional Animal Care and Use Committee of Chongqing Medical University and all methods were performed in accordance with the relevant guidelines and regulations. Group I and group III was injected with 200 µl of sterile PBS per mouse and each mouse of Groups II and IV was injected with 200 μL of bifidobacterium (BF) (1 × 10^8^ bacilli/ml) through the tail vein. After 7 days, each tumor-bearing mouse of group I and group II was injected with 200 μl of sterile PBS through the tail vein, and each tumor-bearing mouse of group III and group IV was injected with 200 μl of CL-NPs via the tail vein. Four groups of tumor-bearing mice were injected with CL-NPs and PBS for 48 hours. The mice were placed in the prone position on the HIFU treatment bed and the tumor site was completely immersed in degassed water. For the four groups of tumor-bearing mice, the acoustic power of HIFU ablation was 150 w, and the ablation time was 5 s. Treatment parameters:the treatment head frequency was 0.94 MHz, the focal length was 145 mm, and the ultrasonic transducer diameter was 220 mm. The gray value in the ablation zone before and after ablation was recorded by Gray Val 1.0 software (Chongqing Haifu Medical Technology Co., Ltd., Chongqing, China). After ablation, coagulative necrosis volume (V) and energy efficiency factor (EEF) were calculated according to the following method: V (mm 3) = (π/6) × length × width × depth; EEF (J/mm 3) = ηPt/V, where η is the focus coefficient of the HIFU transducer (η was set to 0.7 in the instrument), P (W) was the total acoustic power of HIFU, and t (s) was the total treatment time. EEF represents the ultrasonic energy required to ablate a unit volume of a tumor or other lesion. The change of gray value and the volume of coagulative necrosis are proportional to the effect of HIFU ablation, and the EEF value is inversely proportional to the ablation effect of HIFU. One day after HIFU ablation, all tumor-bearing mice were sacrificed. The tumor was completely stripped and cut into 3 mm thick sections. Then, representative necrotic tumor tissues were selected and stained with 2,3,5-triphenyltetrazolium chloride (TTC) solution for 30 min at 37 °C, coagulative necrosis and the value of EEF in the target region were calculated. Another 1 cm^3^ section of tumor tissues was fixed in 4% paraformaldehyde for 24 h, which were embedded in paraffin and used for hematoxylin and eosin (HE) staining.

### Statistical analysis

All datas were expressed as mean ± standard deviation (SD) and all statistical analyses were performed using SPSS software (version 21.0). One way ANOVA was performed for multiple comparison, and student’s t-test were used for intergroup comparison, P < 0.05 was considered statistically significant.

## Data Availability

The datasets generated during and/or analysed during the current study are available from the corresponding author on reasonable request.
